# Feasibility of community at-home dried blood spot collection combined with pooled reverse transcription PCR as a viable and convenient method for malaria epidemiology studies

**DOI:** 10.1186/s12936-022-04239-x

**Published:** 2022-07-14

**Authors:** Dianna E. B. Hergott, Tonny J. Owalla, Jennifer E. Balkus, Bernadette Apio, Jimmy Lema, Barbara Cemeri, Andrew Akileng, Annette M. Seilie, Chris Chavtur, Weston Staubus, Ming Chang, Thomas G. Egwang, Sean C. Murphy

**Affiliations:** 1 Department of Laboratory Medicine and Pathology, University of Washington, 750 Republican St., F870, Seattle, WA 98109 USA; 2 Department of Epidemiology, School of Public Health, University of Washington, Seattle, WA USA; 3 Med Biotech Laboratories, P.O. Box 9364, Kampala, Uganda; 4 Center for Emerging and Re-Emerging Infectious Diseases, University of Washington, Seattle, WA USA; 5 Department of Microbiology, University of Washington, Seattle, WA USA

**Keywords:** *Plasmodium falciparum*, Asymptomatic malaria, Asymptomatic, DBS, At-home

## Abstract

**Background:**

Many *Plasmodium* infections in endemic regions exist at densities below the limit of detection of standard diagnostic tools. These infections threaten control efforts and may impact vaccine and therapeutic drug studies. Simple, cost-effective methods are needed to study the natural history of asymptomatic submicroscopic parasitaemia. Self-collected dried blood spots (DBS) analysed using pooled and individual quantitative reverse transcription polymerase chain reaction (qRT-PCR) provide such a solution. Here, the feasibility and acceptability of daily at-home DBS collections for qRT-PCR was studied to better understand low-density infections.

**Methods:**

Rapid diagnostic test (RDT)-negative individuals in Katakwi District, northeastern Uganda, were recruited between April and May 2021. Venous blood samples and clinic-collected DBS were taken at enrollment and at four weekly clinic visits. Participants were trained in DBS collection and asked to collect six DBS weekly between clinic visits. Opinions about the collection process were solicited using daily Diary Cards and a Likert scale survey at the final study visit. Venous blood and DBS were analysed by *Plasmodium* 18S rRNA qRT-PCR. The number of participants completing the study, total DBS collected, and opinions of the process were analysed to determine compliance and acceptability. The human internal control mRNA and *Plasmodium* 18S rRNA were evaluated for at-home vs. clinic-collected DBS and venous blood to assess quality and accuracy of at-home collected samples.

**Results:**

One-hundred two adults and 29 children were enrolled, and 95 and 26 completed the study, respectively. Three individuals withdrew due to pain or inconvenience of procedures. Overall, 96% of participants collected ≥ 16 of 24 at-home DBS, and 87% of DBS contained ≥ 40 µL of blood. The procedure was well tolerated and viewed favourably by participants. At-home collected DBS were acceptable for qRT-PCR and showed less than a one qRT-PCR cycle threshold shift in the human control mRNA compared to clinic-collected DBS. Correlation between *Plasmodium falciparum* 18S rRNA from paired whole blood and DBS was high (R = 0.93).

**Conclusions:**

At-home DBS collection is a feasible, acceptable, and robust method to obtain blood to evaluate the natural history of low-density *Plasmodium* infections by qRT-PCR.

**Supplementary Information:**

The online version contains supplementary material available at 10.1186/s12936-022-04239-x.

## Background

Over the past decade, substantial progress has been made to reduce malaria morbidity and mortality. However, despite significant investment and robust national anti-malaria programmes, malaria remains endemic in over 85 countries and territories. In 2020, there were 241 million estimated cases and 627,000 deaths [1]. To continue to reduce malaria burden, new control strategies, therapeutics and vaccines are needed. To design the most effective control strategies and accurately assess efficacy of candidate vaccines and therapeutics, it is essential to understand the true malaria burden in endemic populations [2]. Recent analyses show that a large proportion of *Plasmodium* infections in endemic regions are both asymptomatic and low-density [3], so they are not detected by standard field- or clinic-based diagnostic tools. While these infections may not directly lead to clinical illness and death in the individuals who harbour them, they can contribute to onward transmission [4], and may result in immunomodulation [5] that impacts the effectiveness of vaccines and therapeutics. The impact of these infections on malaria transmission dynamics and extent of immunomodulation depends largely on the natural history of infection, but there is a lack of data and limited tools to evaluate parasite dynamics over time in large populations.

The few studies that have assessed natural infection among asymptomatic persons showed that parasite densities were highly dynamic [6–9], and trajectories varied widely between study participants. While these studies are informative, samples were collected in clinics by trained staff, and therefore are limited in size and scope because of logistical and financial constraints. Additionally, infections in these studies were analysed using microscopy [6] or DNA-based PCR [7–9], which are not as sensitive as other molecular methods now available. To better understand how low-density infection impacts malaria transmission and malaria interventions on a large scale, more studies are needed with analytically sensitive methods and in a large variety of endemic areas. Unfortunately, many areas where malaria is endemic have limited access to health care resources. Even in areas with sufficient resources, daily travel to a clinic to provide samples is inconvenient and burdensome. Methods that address these limitations may help resolve some of the gaps in the understanding of low-density infections. Sample collection techniques that are acceptable to participants are needed to allow frequent, accurate, and cost-effective sampling of low-density infections across a variety of settings.

Dried blood spots (DBS) are a convenient, minimally invasive blood collection technique that does not require a clinic or phlebotomist. Proteins and nucleic acids in blood collected on DBS cards are highly stable and can be stored and shipped at ambient temperature, which lowers the cost of shipping and storage and makes them an appealing option in areas with limited or infrequent electricity. DBS have been used for malaria detection in a variety of research studies. While the limit of detection (LoD) for DBS can be lower than whole blood [10], a recent meta-analysis concluded that DBS were non-inferior to venous blood samples for qualitative parasite detection across a variety of settings [11]. Most studies that utilize DBS prepared them from capillary or venous blood drawn in clinic settings, which limits their widespread utilization. However, self-collected DBS samples have been successfully used for the detection of hepatitis C [12] and HIV [13, 14], and for the monitoring of haemoglobin A1c [15] and various drug and vitamin levels [16–21]. This same technique could be applied to study malaria, making it more accessible in areas without health clinics, and allowing for more frequent sampling.

Most previous studies that utilized DBS for parasite detection relied on quantitative PCR of the *Plasmodium* 18S rRNA coding genes [22–31], but DNA-based DBS tests are less sensitive than qRT-PCR for the highly expressed *Plasmodium* 18S rRNAs, which means more infections may be missed by standard DNA-based tests [32]. Ultrasensitive DNA-based tests, such as *var*ATS as well as 18S qRT-PCR assays can detect low levels of parasites from DBS [32–38]. The 18S qRT-PCR methodology was recently adapted to pooled DBS [39], allowing for cost-effective, highly sensitive detection of parasite-expressed 18S rRNA.

Given the ease of DBS collection, stability of DBS analytes over time, and the suitability for pooling, self-collected DBS-based studies might be a feasible way to evaluate and monitor low-density *Plasmodium* infections. Here, the acceptability and feasibility of daily at-home DBS collections as a tool to study *Plasmodium* dynamics was studied over a 28-day period in Katakwi District in Uganda.

## Methods

### Study area

The study was carried out at Med Biotech Laboratories Malaria Clinic in St. Anne Health Center III, Katakwi District in northeastern Uganda. The catchment area of the clinic includes seven villages in Usuk subcounty; participants were recruited from two of these villages. The classical rainy season in Katakwi District is March to November, with marked peaks in April–May and August–October. Transmission is high during the rainy season, peaking in July (72.0 cases/1000 people/month) followed by lower transmission during the dry season (16.2 cases/1000 people in February) [40].

### Study design

This was a longitudinal cohort study to assess the acceptability and feasibility of DBS collection over a 28-day period. Target sample size was 100 adults (18–60 years) and 30 children (8–17 years). The study was approved by the National HIV/AIDS Research Committee (NARC) of the Uganda National Council for Science and Technology (UNCST) (Approval #: ARC 228) as well as the University of Washington (UW) Institutional Review Board (STUDY00009434). All adult participants provided written consent, while children provided assent along with written consent of a parent or guardian, per UNCST guidelines. After providing informed consent, study staff administered a 10-question Assessment of Understanding to potential adult participants (and/or parents of children), and only those who answered at least 80% correctly were permitted to continue with screening and enrollment.

After consenting, prospective participants were evaluated for eligibility and basic demographic information (age, sex, occupation) were collected. Participants answered questions about malaria prevention behaviours and were assessed for malaria signs and symptoms. All data was captured through an interviewer administered questionnaire. Forehead temperature, weight, height, blood pressure and pulse were measured, and all participants were offered a malaria rapid diagnostic test (RDT). Healthy volunteers who met inclusion criteria, including being asymptomatic for Grade 2 or higher malaria-related symptoms and negative for *Plasmodium* parasites by SD Malaria Ag Pf/Pan RDT (Standard Diagnostics Inc, Republic of Korea), were sequentially enrolled into the study until the sample size per each age category was attained. Full inclusion and exclusion are presented in Additional file 1: Table S1.

**Fig. 1:**
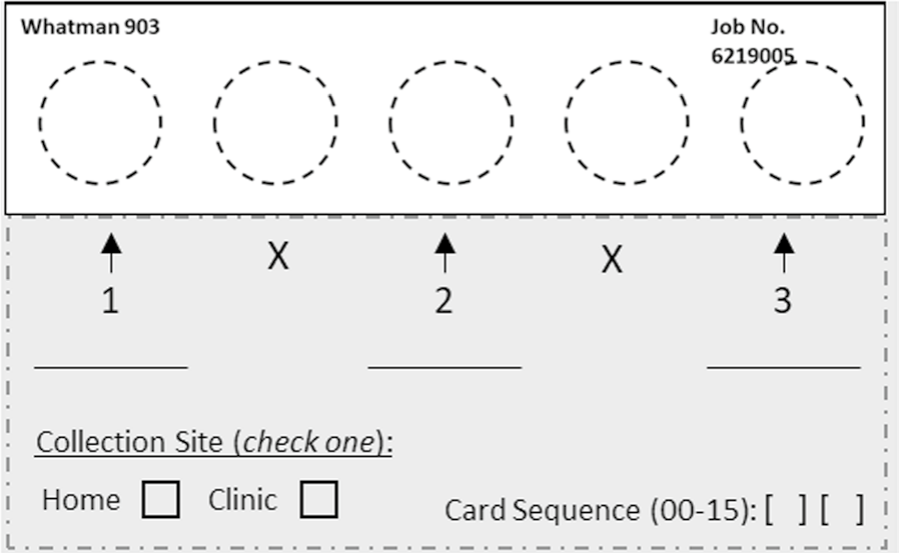
Example of labeled DBS card provided to participants. Numbers and arrows indicated where blood should be spotted

Eligible participants attended an enrollment visit and were then invited to attend four weekly clinic visits and collect a single DBS each day of the week when they did not come into the clinic. They were trained in DBS collection by study staff at enrollment and retrained at subsequent clinic visits if needed. Weekly, participants were provided with all materials to collect at-home DBS for the following week. One DBS card was designed to collect DBS for three days, placed on the first, third, and fifth spot positions on the card (Fig. 1). After DBS collection, participants were asked to fill out a daily Diary Card, indicating in which spot they put the blood, if they had fever or other symptoms, if they were willing to continue, and if they slept under a bed net the previous night. In addition, they were asked to rate the level of pain they felt after the blood prick, on a scale from 0 to 5 (“no pain” to “great pain”). Due to delays in obtaining a version of the Diary Card in the local language at the study start, information on the Diary Card was recorded retrospectively by clinic staff during weekly visits for the first week for all participants, as well as the second week for the first 11 participants enrolled. Once translated versions of the Diary Cards were obtained, participants filled them out at home, and they were reviewed with study staff at weekly visits.

During clinic visits, participants turned in collected DBS cards and received supplies for the following week. In addition, at each visit, two DBS were collected by clinic staff, a venous blood sample was taken, forehead temperature was recorded, and malaria signs and symptoms in the past 24 h and malaria prevention behaviours in the past week were captured through a structured interviewer-administered questionnaire. Any participant with malaria signs and symptoms during the study period was tested using an RDT. Anyone positive by RDT [denoted as RDT(+)] was treated with anti-malarials as per the Ministry of Health-Uganda guidelines [41] and subsequently withdrawn from further DBS study participation. Acceptability of the study was assessed using a 5-point Likert scale questionnaire administered by study staff at the final clinic visit. The questionnaire assessed participants’ feelings on the painfulness of the procedure, ease in collecting DBS, likelihood of collecting DBS for a longer period of time, and whether participants would prefer to come to the clinic over collecting DBS at home. Participants were compensated equivalent to 10 USD for the screening/enrollment visit, and then 5 USD for each week’s completed DBS card and 5 USD for subsequent study visits.

### Sample collection

At each clinic visit, 4 mL of venous blood was drawn from adults and 1 mL from children into K_2_-EDTA vacutainer tubes (BD-Thermo Fisher Scientific Inc., Sweden). Within 24 h, 50 μL of whole blood was transferred into two 2 mL screw top tubes containing 1 mL of pre-aliquoted NucliSENS lysis buffer (bioMérieux, Marcy-l’Etoile, France) and gently mixed by inverting the tube. The samples were immediately stored at − 20 °C for a maximum of 7 days at the field site before transfer to the central laboratory, where they were stored at < − 70 °C. An additional 1 mL (adult) or 0.5 mL (child) of whole blood was transferred to a 2 mL tube and stored at ≤ − 20 °C. The remaining blood was centrifuged, and the plasma supernatant was transferred to clean tubes and stored at ≤ − 20 °C.

DBS were collected on Protein Saver 903 DBS cards (Global Life Sciences Solutions, Buckinghamshire, UK) using automatic lancets (Accu-Chek Safe-T-pro Uno 1.5 mm/MM 28G, Roche, Germany). Cards were stored in gas-impermeable plastic bags, with desiccators and a moisture indicator. After collection, cards were dried at the clinic or participant’s home for at least four hours but no more than 12 h, and then stored at room temperature. Participants were instructed to dry cards out of direct sunlight or heat. DBS cards were stored (5–6 months) and shipped at ambient temperature until processing.

### Laboratory methods

Prior to cutting, the volume of blood on each spot to the nearest 10 μL was estimated by comparing the spot to a Protein Saver card with known volumes of pipetted blood. DBS spots were then laser cut using contact-free methods [42], deposited into 2 mL NucliSENS lysis buffer and incubated at 55 °C for 30 min as previously described [39]. A maximum of three pools per participant were needed to test 28 DBS. Standard pools were constructed by combining 0.2 mL aliquots of DBS lysis buffer samples from each of 7–10 individual DBS from the same participant (within-participant pooling). In some cases, pools were derived from 2 to 6 samples, with lysis buffer added to achieve a minimum total volume of 1.3 mL. RNA was extracted from 1 mL of the pooled lysate and eluted into 88μL of elution buffer by the Abbott m2000*sp* system using the mSample RNA preparation kit (Abbott Molecular, Des Plaines, USA). A total of 15 µL of the extracted RNA was combined with 35 µL of SensiFAST Probe Lo-ROX One-Step Kit mastermix (Meridian Bioscience, Cincinatti, USA) and subjected to qRT-PCR on the Abbott m2000*rt*. The qRT-PCR primers and probes targeted the *Plasmodium falciparum* (*Pf*) 18S rRNA (Forward: PfDDT1451F21: 5′-GCGAGTACACTA TATTCTTAT-3′; Reverse: PfDDT1562R21: 5′-ATTATTAGTA GAACAGGGAAA-3′; Probe: 5′-[6-FAM]-ATTTATTCA GTAATCAAAT TAGGAT-3′ [Black Hole Quencher 1 PLUS]; LGC BioSearch Technologies, Petaluma, USA), pan-*Plasmodium* 18S rRNA (Forward: PanDDT1043F19: 5′-AAAGTTA[+ A]GG GA[+ G][+ T]GAAGA-3′, Reverse: PanDDT1197R22: 5′-AA[+ G]ACTTTGA TTTCTC[+ A]TAAGG-3′; Qiagen, Hilden, Germany; Probe: 5′-[CAL Fluor Orange 560]-ACCGTCG TAATCTTAACCATAAACTA[T(Black Hole Quencher-1)] GCCGACTAG-3′[Spacer C3]; LGC BioSearch Technologies), and the human TATA binding protein (TBP) mRNA control (Forward: 5′-GATAAGAGA GCCACGAACC AC-3′; Reverse: 5′-CAAGAACTTAGCTG GAAAACCC-3′; Probe: 5′-[Quasar 670]-CACAGGAGCCAA GAGTGAAGAACAGT-3′[Black Hole Quencher-2]; LGC BioSearch Technologies) as previously described [43], except with thermocycling conditions of 10 min at 45 °C, 2 min at 95 °C, followed by 40 cycles of 5 s at 95 °C and 35 s at 54 °C. Initial runs for DBS were conducted using within-participant pools of up to 10 samples per pool. If the pool was negative, all samples were reported as negative. If the pool was positive (using a threshold of ≥ 2 estimated parasites/mL whole blood), samples were deconvoluted and re-tested individually. For lysed venous blood samples (already containing 50 µL of blood in 1 mL of lysis buffer as noted above), samples were thawed, 1 mL of additional fresh lysis buffer was added, and then 1 mL of the total lysate was extracted and processed for qRT-PCR as described for individual DBS.

### Statistical analyses

To analyse the feasibility of DBS collection as a tool to evaluate the daily dynamics of *Plasmodium* infections, compliance, acceptability, and quality of the DBS collected were assessed. Analyses were stratified by age category (adults or children) and sex (in adults only). Study data was captured on paper case report forms and then input and managed using REDCap electronic data capture tools hosted at UW [44, 45]. Verification of data transcription by a second reviewer was performed on 10% of all entries. All statistical analyses were performed in RStudio v12.5033 (Boston, MA).

Characteristics of screened and enrolled participants were summarized using descriptive statistics. The number of participants who withdrew each week and reasons for withdrawal are presented. Compliance was evaluated by summarizing the number of at-home DBS that were collected throughout the study period. As there was no standard for what was expected, categories were created based on what were assumed to be reasonable patterns of compliance: ‘excellent’ compliers were those that missed no more than two spots during the entire study (22–24 spots), ‘good’ compliers missed no more than 1–2 spots per week or one whole week of collection (16–22 spots), ‘fair’ compliers missed 2–3 spots per week or two collection weeks (11–14 spots), and ‘poor’ compliers missed more than half of the samples each week (< 11 spots). The number and proportion of excellent and good compliers (≥ 16 DBS) and fair and poor compliers (< 16 DBS) are presented. Individuals who were withdrawn because they developed clinical malaria prior to Day 18 of the study were excluded, as they did not have the opportunity to collect at least 16 samples during their participation.

Acceptability was assessed through descriptive analyses of the final visit opinion survey answers as well as an evaluation of reported pain scores over time, collected via Diary Card. Mean daily pain scores and 95% CI were calculated and plotted to assess the overall pattern throughout the study. Weekly average pain scores were compared using a paired t-test. Mean values were summarized based on available data and missing dates were not imputed. As a secondary analysis, patterns of pain over time were categorized among individuals who had complete pain data reported, defined as a pain rating for each blood spot that was collected.

Quality of the DBS was evaluated by summarizing the volume of blood collected by participants each day, and the presence of the TBP human mRNA quality control marker in each sample. The number and proportion of samples in each 10 µL volume category (10–50 µL) during each week of collection were calculated and compared as a measure of quality of DBS collection. To evaluate the integrity of the DBS and analyse whether there was possible degradation of samples collected, the number and proportion of total spots that failed quality control [had no TBP cycle threshold (CT)] were summarized. For each individual with deconvoluted samples, the mean TBP CT value and standard deviation (SD) of all their samples were calculated. The proportion of individuals with TBP SD < 1 cycle and > 1 cycle was summarized. An ordinal linear regression model was run to assess any relationship between sample volume and TBP CT, and pairwise t-tests between TBP CT values of the various volume categories were conducted. To analyse whether samples collected at home may have been of poorer quality than those collected at the clinic, the mean TBP CT values of clinic-collected DBS vs. at-home-collected DBS samples were compared using a student’s t-test. Mean TBP CT values and 95% CI of samples collected on the first day of each week were compared to those collected on the final day of sampling each week using a student’s t-test to determine whether those collected earlier in the week and, therefore, exposed to more air, may have been subject to further degradation.

Finally, to assess the accuracy of DBS as a parasite detection tool for low-density infections, the correlation between estimated log_10_ *Pf* 18S rRNA copy numbers detected from paired clinic-collected venous and DBS samples, collected from the same person on the same study day, was estimated using Pearson’s correlation coefficient. Samples with copy numbers below the DBS LoD (20 estimated parasites/mL) were classified as not detected. As not all DBS contained 50 μL of blood, which is what the qRT-PCR quantification curve is calibrated to, additional sensitivity analyses were conducted using volume-adjusted *Pf* 18S rRNA copy numbers for DBS, as well as restricting analyses to include only DBS with 50 µL of blood. A conversion factor was calculated by dividing 50 μL by the actual estimated volume of the spot, and then adding the log_10_ value of the conversion to the estimated copy number that assumed a 50 μL spot.

### Cost savings estimates

To determine the cost savings of utilizing within participant pooling, calculations were made to compare the costs of analysing the samples with and without pooling. The without pooling estimate was determined by calculating the number of PCR runs that would have been necessary to test all DBS collected, and then compared to the PCR runs that were actually done using pooling and deconvolution. Costs for RNA extraction, qRT-PCR reagents and material cost, and labor were considered. Each pooling and deconvolution set was estimated at a cost of 1100 USD in reagents and material and 4.5 person-hours. Non-pooled runs were estimated to cost 1070 USD in reagents and materials per run and utilize 3.5 person-hours.

## Results

### Study enrollment and demographics

**Fig. 2:**
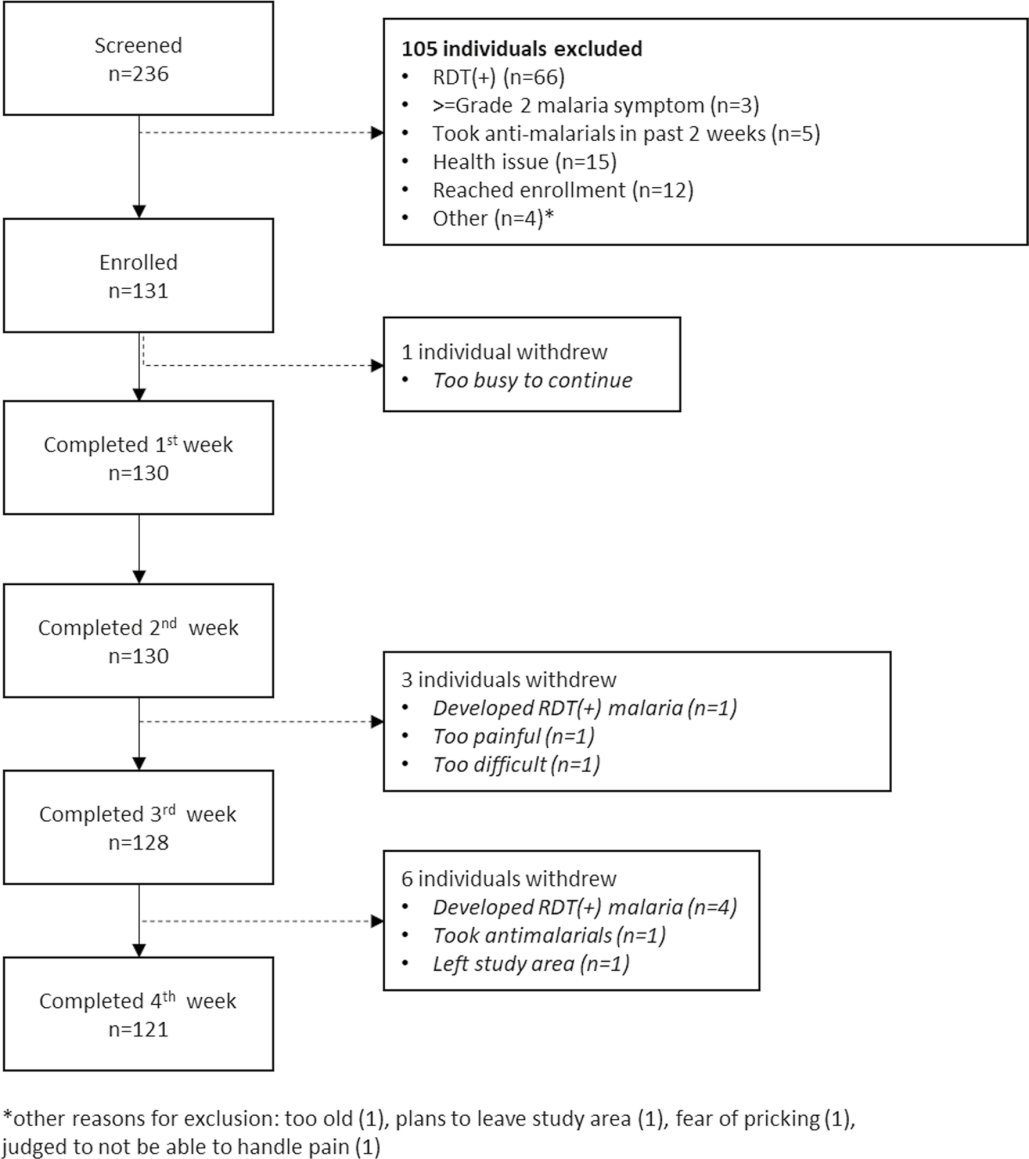
Study disposition showing number screened, enrolled, and continuing in the study by week

This study recruited and enrolled 102 adults (median age 33 years; range 18–59) and 29 children (median age 13 years, range 8–17) from Opoyongo and Oleroi villages between April 9 and April 22, 2021. The final study visit occurred on May 20, 2021. Characteristics of those screened and enrolled and study flow are shown in Table 1 and Fig. 2, respectively. In both adults and children, a higher proportion of males were screened than enrolled, and a lower proportion of those screened reported sleeping under a bed net the previous night compared to those enrolled. There were no notable differences in other demographic or malaria behaviour characteristics between those enrolled and not enrolled. The main reason for exclusion was being RDT(+) at screening: 37 adults (22.2%) and 29 children (42.0%) tested positive for *P. falciparum* by RDT during the screening visit**,** for an overall prevalence of RDT(+) malaria of 28% (95% CI 22.6, 34.0) in the screened population.

**Table 1:** Demographic, health, malaria prevention behaviours and malaria symptoms in adults and children screened and enrolled for the 28-day at-home DBS feasibility study in Katakwi, Uganda

	Children		Adults		Overall	
	Not enrolled	Enrolled	Not enrolled	Enrolled	Not enrolled	Enrolled
	(N = 40)	(N = 29)	(N = 65)	(N = 102)	(N = 105)	(N = 131)
Demographics and health						
Age (years)	12 (± 2.6) [8.0, 17]	13 (± 2.6) [8.0, 17]	34 (± 13) [18, 64]	33 (± 12) [18, 59]	25 (± 15) [8.0, 64]	29 (± 13) [8.0, 59]
Male sex (%)	24 (60.0%)	12 (41.4%)	44 (67.7%)	42 (41.2%)	68 (64.8%)	54 (41.2%)
Weight (kgs)	34 (± 9.6) [19, 63]	39 (± 11) [22, 59]	57 (± 6.9) [41, 73]	58 (± 8.7) [41, 85]	48 (± 14) [19, 73]	54 (± 12) [22, 85]
Height (cm)	34 (± 9.6) [19, 63]	39 (± 11) [22, 59]	170 (± 23) [75, 200]	170 (± 16) [82, 200]	170 (± 25) [54, 200]	170 (± 20) [50, 200]
Febrile at screening	0 (0%)	0 (0%)	0 (0%)	0 (0%)	0 (0%)	0 (0%)
Village						
Opoyongo	32 (80.0%)	19 (65.5%)	33 (50.8%)	45 (44.1%)	65 (61.9%)	64 (48.9%)
Oleroi	8 (20.0%)	10 (34.5%)	32 (49.2%)	57 (55.9%)	40 (38.1%)	67 (51.1%)
Occupation						
Peasant farmer	2 (5.0%)	0 (0%)	60 (92.3%)	96 (94.1%)	62 (59.0%)	96 (73.3%)
Not applicable	37 (92.5%)	27 (93.1%)	5 (7.7%)	5 (4.9%)	42 (40.0%)	32 (24.4%)
Other	1 (2.5%)	2 (6.9%)	0 (0%)	1 (1.0%)	1 (1.0%)	3 (2.3%)
Malaria prevention behaviours						
Slept under bednet previous night	32 (80.0%)	25 (86.2%)	55 (84.6%)	94 (92.2%)	87 (82.9%)	119 (90.8%)
Slept under net in past week	32 (80.0%)	25 (86.2%)	56 (86.2%)	94 (92.2%)	88 (83.8%)	119 (90.8%)
House has open eaves	39 (97.5%)	29 (100%)	64 (98.5%)	102 (100%)	103 (98.1%)	131 (100%)
Principal wall material						
Mud	38 (95.0%)	29 (100%)	64 (98.5%)	99 (97.1%)	102 (97.1%)	128 (97.7%)
Brick	2 (5.0%)	0 (0%)	1 (1.5%)	3 (2.9%)	3 (2.9%)	3 (2.3%)
House sprayed with insecticide in past 6 months	0 (0%)	0 (0%)	0 (0%)	0 (0%)	0 (0%)	0 (0%)
House smeared with insecticide-treated soil	0 (0%)	0 (0%)	0 (0%)	0 (0%)	0 (0%)	0 (0%)
Spends more than 2 h outside of house between dusk and dawn	40 (100%)	29 (100%)	65 (100%)	101 (99.0%)	105 (100%)	130 (99.2%)
Grade 2 or higher malaria symptoms reported						
Chills/rigors	1 (2.5%)	0 (0%)	0 (0%)	0 (0%)	1 (1.0%)	0 (0%)
Headache	0 (0%)	0 (0%)	0 (0%)	0 (0%)	0 (0%)	0 (0%)
Fatigue/malaise	0 (0%)	0 (0%)	0 (0%)	0 (0%)	0 (0%)	0 (0%)
Myalgia	0 (0%)	0 (0%)	0 (0%)	0 (0%)	0 (0%)	0 (0%)
Low back pain	0 (0%)	0 (0%)	1 (1.5%)	0 (0%)	1 (1.0%)	0 (0%)
Vomiting	0 (0%)	0 (0%)	0 (0%)	0 (0%)	0 (0%)	0 (0%)
Diarrhea	0 (0%)	0 (0%)	0 (0%)	0 (0%)	0 (0%)	0 (0%)
Abdominal pain	0 (0%)	0 (0%)	0 (0%)	0 (0%)	0 (0%)	0 (0%)
Arthralgia	0 (0%)	0 (0%)	0 (0%)	0 (0%)	0 (0%)	0 (0%)
Chest Pain	0 (0%)	0 (0%)	0 (0%)	0 (0%)	0 (0%)	0 (0%)

### Compliance

Ten of the 131 participants (8%) did not complete the full study. One adult withdrew in the first week, citing being too busy to continue. Three participants withdrew between the second and third weeks of collection; two cited the procedure as too painful or too difficult, and one had RDT(+) malaria. Six participants were withdrawn during the final week of collection; four were withdrawn because they developed symptomatic RDT(+) malaria, one left the study area, and one took anti-malarial medications (Fig. 2 and Table 2).

**Table 2:** Summary of compliance in dried blood spot collection over the 28-day study period for adults and children

	Adult females	Adult males	Children	All
Excellent or Good compliers (≥ 16 samples collected)^a^	60/60 (100%)	36/39 (92%)	27/28 (96%)	123/127 (96%)
Fair or poor compliers (< 16 samples collected)^a^	0/60 (0%)	3/39 (8%)	1/28 (4%)	4/127 (3.1%)
Developed RDT(+) *falciparum* malaria during study^b^	1/60 (1.6%)	2/42 (4.8%)	2/29 (6.9%)	5/131 (3.8%)
Discontinued in week 1^b^	0/60 (0%)	1/42 (2.4%)	0/29 (0%)	1/131 (0.8%)
Discontinued in week 2^b^	0/60 (0%)	0/41(0%)	0/29 (0%)	0/130 (0%)
Discontinued in week 3^b^	0/60 (0%)	1/40 (2.5%)	1/29 (3.5%)	2/129 (1.6%)
Discontinued in week 4^b^	1/59 (1.7%)	1/38 (2.6%)	0/26 (0%)	2/123 (1.6%)

A total of 24 home blood spots were possible for individuals that completed the entire study

^a^Denominator includes DBS samples received at University of Washington laboratory and excludes 1 individual (adult male) who developed malaria before Day 18 of study

^b^Denominator includes all participants enrolled in study. Excludes any individuals who discontinued because of malaria in given week

Of the 131 enrolled individuals, DBS cards were received at the University of Washington (UW) for 100/102 adults and 28/29 children and are included in the assessment of compliance. One individual developed malaria prior to Day 18. Of the 126 individuals who did not develop RDT(+) malaria by Day 18 of the study, 96% of individuals were good or excellent compliers (Table 2). Only one adolescent (4%) and three adults, whom were all males, collected < 16 samples during the study.

### Acceptability

**Fig. 3:**
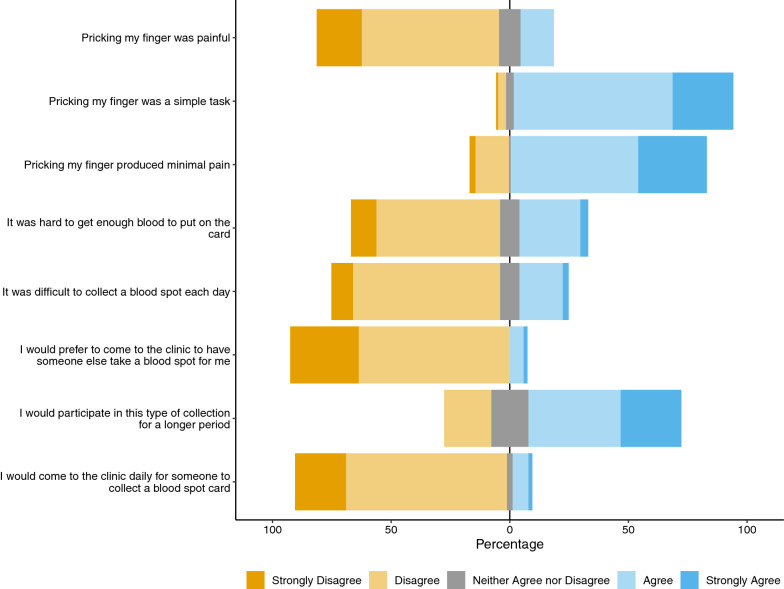
Responses from 123 participants to the 5-point Likert scale questionnaire soliciting opinions about the DBS procedures

Acceptability of the study procedure was high (Fig. 3). Almost all (93%) participants indicated that pricking their finger was a simple task, and only 7% of participants indicated they would prefer to come to the clinic to have someone else collect the DBS for them. Most participants viewed the process positively, though 21% said they would not participate in this type of collection for a longer period; 10 children and 14 adults. Five children provided additional explanation, all citing the pain or difficulty of the process as their reasoning for not wanting to continue. Survey reliability was determined by congruency between questions designed to elicit opposite responses from participants. For example, when comparing the responses to “pricking my finger was a simple task” and “it was difficult to collect a blood spot each day”, congruency between questions was 81% (95% CI 73–87%). Congruency was 75% (95% CI 67–82%) when comparing responses to “pricking my finger produced minimal pain" and “pricking my finger was painful.”

**Fig. 4:**
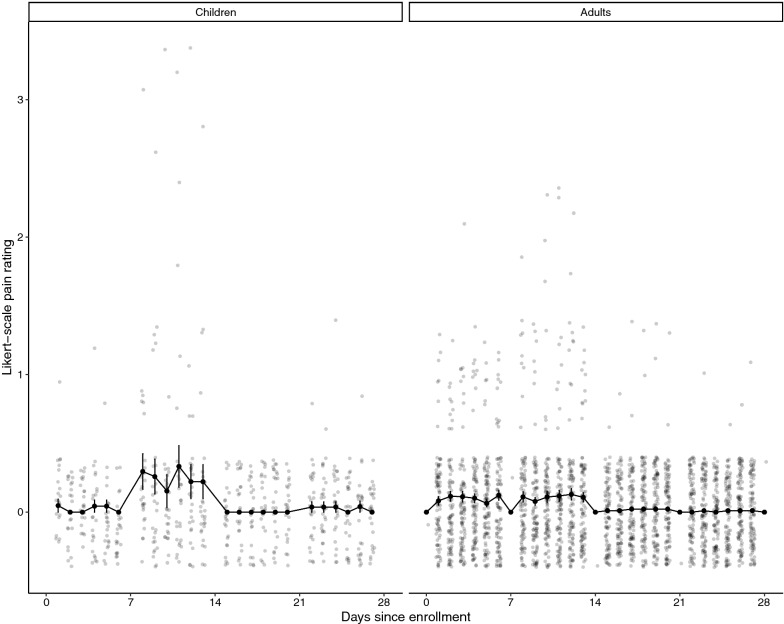
Reported pain of finger prick procedure by day of study for children (n = 29) and adults (n = 102). 0 = no pain to 5 = great pain. Individual values are shown as opaque scattered points, and the daily mean with 95% CI are presented as solid black dots and lines

**Fig. 5:**
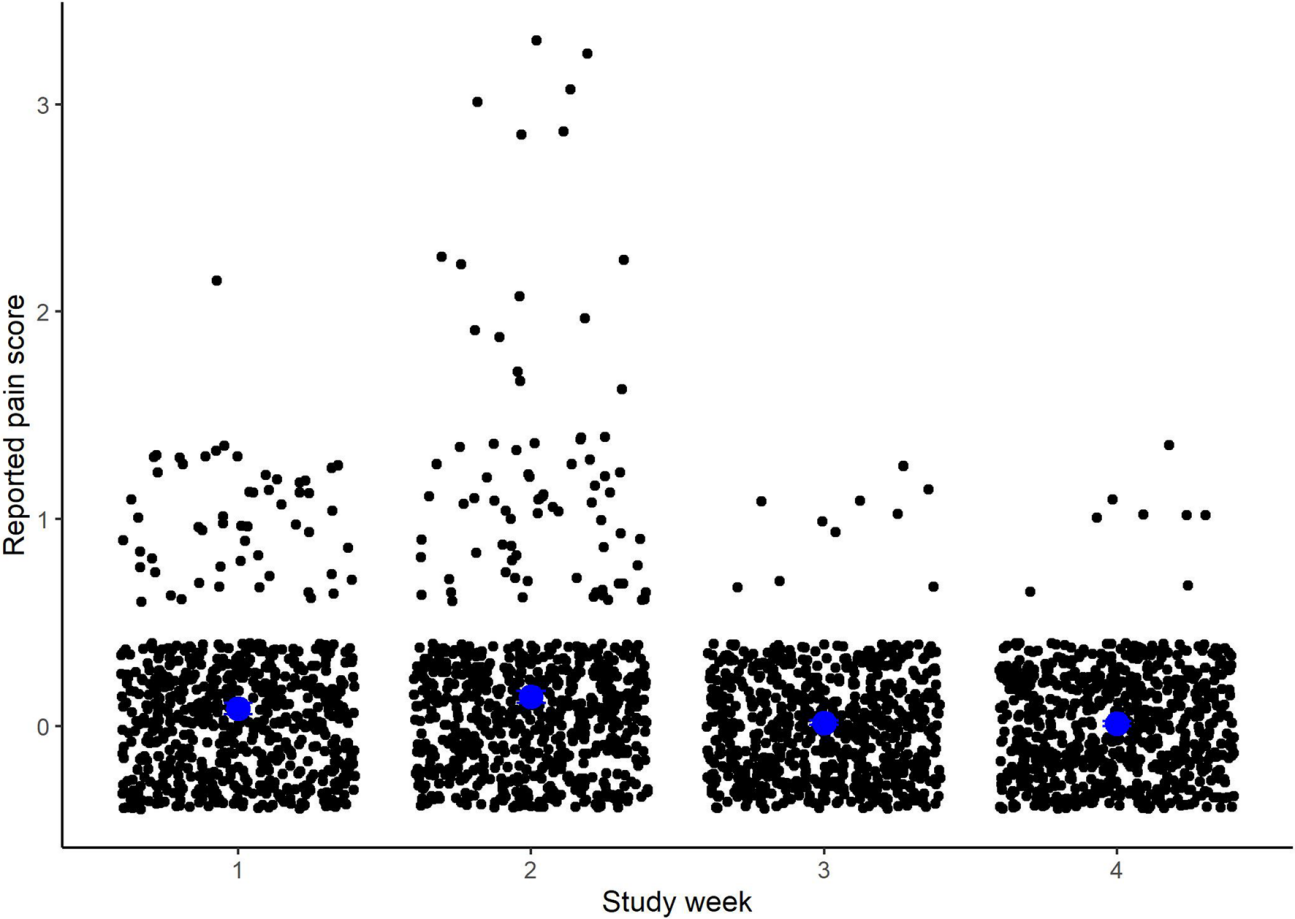
Reported pain by study week. Blue dots represent the mean weekly score, black dots represent individual responses

The mean pain score and 95% CI by day and age group is shown in Fig. 4, and the average weekly pain scores are presented in Fig. 5. Most participants reported little to no pain from the procedure throughout the study duration. While the average weekly pain scores were always below 1, pain scores were statistically significantly greater in week 2 compared to weeks 1 (0.14 vs. 0.08, p = 0.004), 3 and 4 (0.08 and 0.08, respectively). Complete data reporting for pain was available for 82 participants (62%), 67 adults and 15 children. Among those, two-thirds of participants reported no pain from the finger prick during the entire study. Adult males were more likely to report no pain compared to adult females and children (80% vs. 59% and 60%, respectively). Among those who reported pain, 12% only reported pain in the first two weeks of the study, while an additional 15% reported pain during the second study week only.

### DBS quality

The volumes of 2955 individual home-collected DBS were estimated. Overall, 73% had ≥ 50 μL, and 87% of samples had ≥ 40 μL of blood, volumes considered optimal for the qRT-PCR assay (Table 3). There were 44 individuals (35%) who had ≥ 40 μL blood on every spot collected during the study period. Blood volume was lowest in week one when only 65% of samples had ≥ 40 µL of blood. In subsequent weeks, nearly all DBS were of sufficient volume.

**Table 3:** Number and proportion of total samples that were estimated to certain volumes by study week

Spot volume (µL)	Week 1 (n = 747)	Week 2 (n = 756)	Week 3 (n = 743)	Week 4 (n = 708)	All (n = 2955)
≥ 50	357 (0.48)	589 (0.78)	613 (0.83)	612 (0.86)	2171 (0.73)
40	126 (0.17)	108 (0.14)	108 (0.15)	78 (0.11)	420 (0.14)
30	114 (0.15)	38 (0.05)	21 (0.03)	15 (0.02)	189 (0.06)
20	87 (0.12)	16 (0.02)	1 (0.00)	3 (0.00)	107 (0.04)
10	41 (0.05)	3 (0.00)	0 (0.00)	0 (0.00)	44 (0.01)
< 10	22 (0.03)	2 (0.00)	0 (0.00)	0 (0.00)	24 (0.01)

There were 82 individuals who had *Plasmodium* parasites detected at some point during the 28-day follow-up period. From these individuals, there were 1780 individual DBS that were deconvoluted and available for the analysis of human TBP mRNA quality. All individually-tested samples had TBP detected. The mean TBP CT for the DBS was 34.99 ± 1.22 cycles (range: 28.69–39.23). Individually, the SD among all samples collected was less than one cycle for 60 participants (74%), while 21 participants (26%) had SD of greater than one cycle. One participant had only a single sample and, therefore, there was no SD. The maximum SD of any participant was 1.62 cycles.

**Fig. 6:**
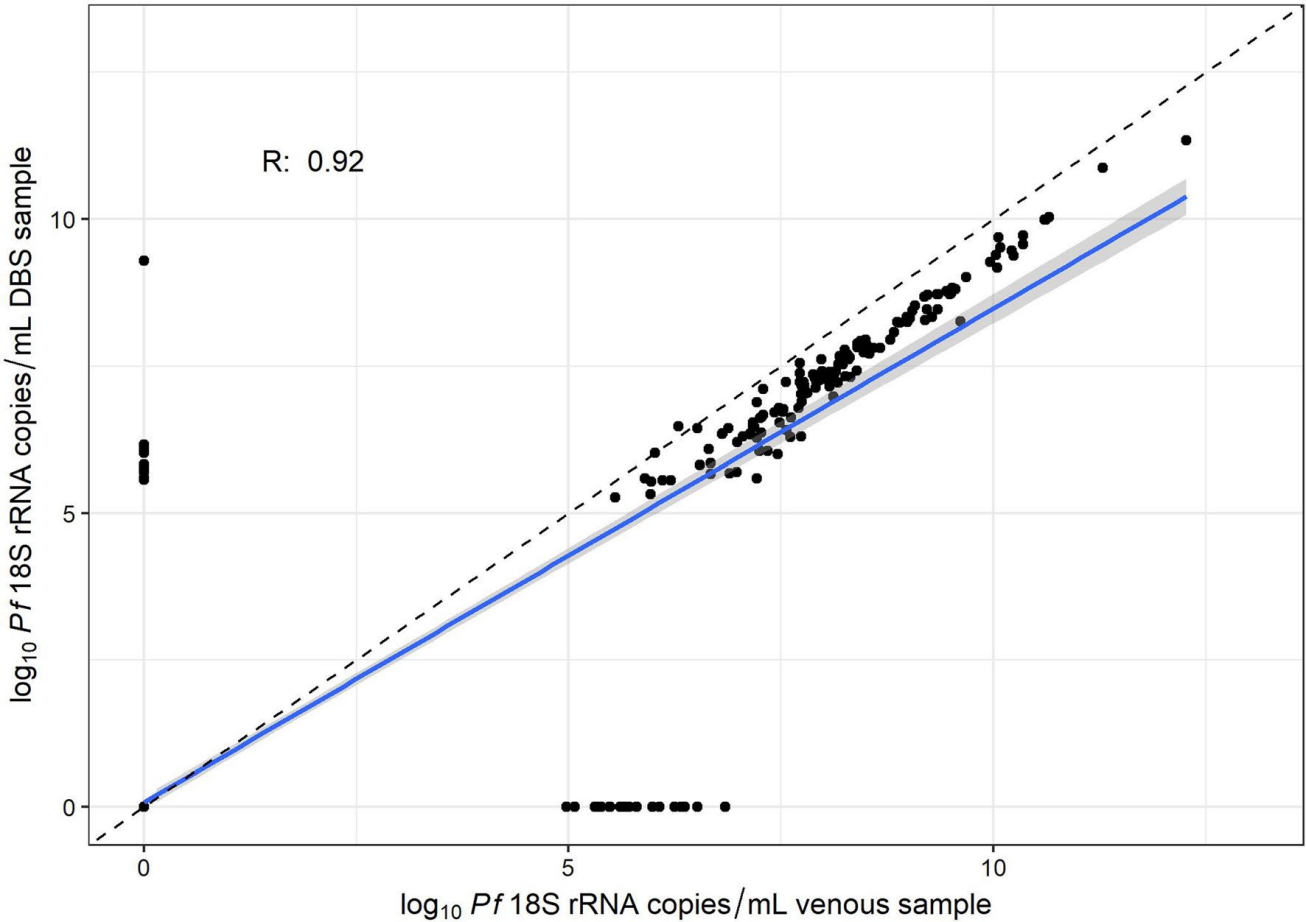
Distribution of TBP cycle threshold (CT) values by estimated volume of blood collected on the filter paper for dried blood spots (DBS) collected at home (left panel) and at the clinic (right panel)

There was no difference in TBP CT values by volume in clinic collected samples, but there was a small, but inconsequential, decrease in CT value of 0.01 cycles per each additional 10μL of blood on the home collected DBS (95% CI − 0.16, − 0.004). However, pairwise comparisons of TBP CT values of various volumes showed no significant difference in CT values of the home collected samples between 50, 40, 30 and 20 μL. Significant differences were seen between CT values of DBS with 10 µL and < 10 μL and all higher volumes. There were no differences in pairwise comparisons of the clinic collected spots, in which no spot had < 30 µL of blood (Fig. 6).

Among deconvoluted samples, there were 314 spots collected at the clinic and 1466 spots collected at home. The TBP CT for clinic-collected spots was slightly earlier than for at-home collected spots (mean clinic-collected TBP CT 0.85 cycles earlier than at-home DBS, 95% CI 0.71, 0.98). When comparing DBS collected on the first day of weekly home collection (n = 244) to the last day of weekly home collection (n = 244), there was no difference in mean TBP CTs in these samples (35.15 vs. 35.15 cycles), suggesting that samples stored longer at home and removed more frequently from bags did not degrade any more than those collected at the end of each week. There was also no difference in the average TBP CT values of samples collected during each week of the study (Additional file 2: Table S2). In-clinic collected DBS had average TBP CTs that were 2.59 cycles later than for venous blood (95% CI 2.43–2.72 cycles), and there was moderate correlation between the paired sample types (Pearson’s R = 0.47), indicating that there is some loss of the human mRNA control target on DBS compared to venous blood.

**Fig. 7:**
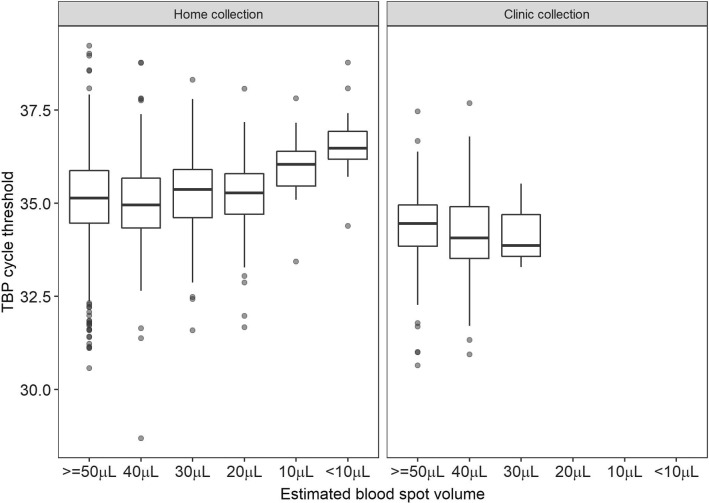
Correlation between log_10_ copy numbers of *P. falciparum* parasites estimated by paired venous and DBS samples collected from the same participant on the same day in Katakwi District, Uganda at weekly clinic visits. The blue line and shaded gray areas is the estimated linear regression line and 95% CI, respectively, between the two estimates. The dashed line represents a slope of 1

There were 619 paired venous blood and DBS samples taken at the clinic from 128 participants. There was very high correlation between the log_10_ *Pf* 18S rRNA copy numbers from paired DBS and venous blood samples (Pearson’s R = 0.93) (Fig. 7). Of the 617 sample pairs, 136 were positive in both venous blood and DBS samples (median log_10_ *Pf* 18S rRNA copy number 8.1 for venous blood [IQR: 7.2–9.0] and 7.3 for DBS [IQR 6.5–8.3]), 453 were negative in both samples, 11 were positive by DBS but negative in venous blood, and 19 were positive in venous blood and negative in DBS. Amongst the 11 DBS-positive/venous-negative discrepant samples, the median log_10_ *Pf* 18S rRNA copy number was 5.8 [IQR: 5.7–6.1]. Amongst the 19 DBS-negative/venous-positive discrepant samples, the median log_10_ *Pf* 18S rRNA copy number was 5.7 [IQR: 5.5–6.2]. There was no difference in correlation when restricting analyses to DBS with 50 µL of blood or when using volume-adjusted copy numbers for DBS samples (Additional file 3: Fig. S1), suggesting that this technique is highly robust, even when the target volume of blood is not obtained.

### Cost analysis of pooling

Overall, 3597 DBS were collected over the study period. Using within-participant pooling, 51 PCR runs were performed, and 1780 individual samples were deconvoluted. Compared to testing all 3597 DBS individually, it was estimated that the within-participant pooling strategy saved an estimated 15800 USD in materials and 9550 USD in labour costs.

### Discussion

This study demonstrates that daily, at-home DBS collection is a feasible and acceptable method to study the natural history of low-density *Plasmodium* infections over time. The within-participant pooling strategy also provided substantial cost savings. In addition to this analysis on the feasibility and acceptability of at-home DBS for malaria studies, the dynamics of these asymptomatic infections provided important epidemiological insights and will be the subject of another forthcoming publication.

Compliance with at-home DBS collection was extremely high. Only three (2%) participants withdrew from the study early due to pain or inconvenience of the collection procedures. Among those who stayed in the study, there was a high rate of compliance, and 85% of participants collected all DBS during the study period. Compliance in this study is similar or higher than compliance in previous studies that looked at longitudinal dynamics through clinic sampling. In a study of adult males in Mali who were sampled two or three times a day for 12–13 consecutive days, 98.5% of all planned samples were collected [6]. In a study of afebrile Mozambican men, 28% of participants missed one follow-up visit at the clinic [8]. Both of these studies were either for a shorter overall duration or required fewer samples over the same time period. It is a strength of the current study technique that there was such high compliance with the longer time and frequency of sampling. About one-third of participants in the current study indicated they would not want to continue in daily sampling for a longer period of time, suggesting that rates of discontinuation may be greater if such studies were extended for a longer time. Children were more likely than adults to indicate they did not want to continue, and among those that provided a reason, the pain or difficulty of the process deterred longer participation. Additional qualitative and quantitative analyses are needed to determine the optimal sampling frequency and duration for different age groups. Compliance with home collection may also decrease in a surveillance setting in which participants are not compensated or given other indirect benefits, such as health checks, for participation. Further community-based research, sensitization, and strategies will be needed to successfully implement home collection on a larger scale.

Participants found the at-home sampling to be a simple and low-pain process; no participant reported pain > 3 on a 5-point Likert scale, and the average weekly pain score was always < 1. The highest pain scores were reported in week 2 of the study. There are several possible explanations for this. First, several participants had to be retrained after the first week of blood collections because there was insufficient blood on their week 1 DBS cards. Insufficient blood can be a result of not pricking the finger deeply enough or not massaging the finger sufficiently to get blood. Therefore, it is possible that participants were not pricking themselves correctly during week one, and when they were retrained in proper technique, there was greater penetration and as a result, slightly more pain. Another explanation for the heightened pain in week 2 could be because participants were pricking their finger for the second time. If the prick site had not fully healed, it may have been more painful the second time. However, if this were the explanation, it would have been expected that pain scores remained increased through weeks 3 and 4 when additional repeat pricking was performed, but that was not reported. Finally, it is possible that the increased pain scores in week 2 are a result of reporting biases. Information on pain for week 1 was captured retrospectively at the clinic. It is possible that participants were hesitant to report pain to study staff because they wanted to continue in the study and, therefore, pain scores in week 1 reflect social desirability bias more than true reflection of the pain of the procedure. Even if there was some bias in the reporting, the overall pain scores throughout the study were low, and pain did not seem to be a barrier to completing the 28-day collection for most participants.

In addition to being feasible to collect, our analyses showed that the DBS collected were of high quality and produced comparable readings to venous blood collections. Comparison of mean human TBP mRNA CTs from DBS collected at the clinic vs. home showed a small reduction in mRNA signal in at-home collected DBS, but both at-home and in-clinic DBS were sufficient to obtain quality qRT-PCR results. Unsurprisingly, the TBP CT values in venous blood samples were earlier than in the DBS collected in-clinic on the same day. However, there was minimal difference seen in the quantification of *P. falciparum* parasites in these samples, supporting the high stability of the 18S rRNA *Plasmodium* target in the samples [39, 46]. Similar results have been seen in other studies comparing venous to DBS samples, and a recent meta-analysis of seven such studies concluded there was no qualitative difference in parasite events detected from DBS compared to whole blood [11]. Notably, the one study in the analysis that also used RT-PCR for 18S rRNA had very high agreement between DBS and whole blood [34]. Studies that used DNA-based 18S PCR tended to have fewer samples identified with DBS compared to whole blood, which may be due to loss of DNA during the extraction process, as recently demonstrated in a systematic comparison of the two sample types [10]. Amongst the *P. falciparum* 18S rRNA discrepant samples (DBS-positive/venous blood negative and vice versa) seen in the current study, most discrepant samples were in the low parasite density range whereby discrepancies could be explained by the stochasticity of rare sampling events. The qRT-PCR approach was recently shown to reliably detect a single parasite in each 50 µL sample when the Poisson distribution-described sampling limitations were overcome [47]. Given the collection, storage, and shipping benefits of DBS over venous blood, the current analysis adds to the evidence that there is little quality difference in the results from each sample type.

Surprisingly, there was no difference in the quality of sample, as measured by human TBP mRNA, when comparing DBS collected at the beginning of a sample week to those collected at the end. Home collected samples, especially the first spot on each card, were subjected to greater handling, removal from desiccant, and exposure to the air than samples collected at the end of the week or at the clinic. Therefore, one might expect more degradation in these samples. Since this was not observed, the data supports a strategy whereby a single DBS card can be used to reliably collect multiple samples at different points in time without sacrificing the quality of the sample. Samples from this study were analysed 6–8 months after collection, and therefore, it is possible that there may be further degradation of the human TBP mRNA and *P. falciparum* 18S rRNA in samples that are stored longer before processing. Current stability studies are ongoing to assess stability over longer time periods that will inform how long samples can sit before being processed.

This study was the first to show that self-collected DBS spots can be used to quantitatively study *Plasmodium* infections over time. The simplicity, stability, and cost-effectiveness of this method will allow for large-scale evaluations of low-density infections in malaria-exposed populations. Home DBS collection overcomes several logistical difficulties encountered in previous studies because such DBS do not require clinic staff nor travel time for participants. Participants in this study overwhelmingly preferred at-home collections to clinic collections, and several of them commented on the convenience and time saving nature of the home collection approach. Home collection could be made even more convenient if participants could either mail in samples, as has been done with previous self-collected DBS for other diseases [13], or if DBS could be collected by village health workers or other Ministry of Health networks that work routinely in communities. Thus, the use of DBS has the potential to expand malaria surveillance into areas that are typically hard to reach due to lack of health infrastructure, to improve clinical trial designs, and to deeply enhance our understanding of the natural history of low-density *Plasmodium* infections.

## Conclusions

At-home DBS collection for analysis by qRT-PCR using within-participant pooling is a feasible, cost-effective strategy to study dynamics of low-density *Plasmodium* infections. This technique should be utilized to improve the current understanding of the contribution of low-density parasite infections to the infectious reservoir, and the possible impact of these infections on therapeutic and vaccine efficacy studies.

## Supplementary Information

**Additional file 1: Table S1. **Full inclusion and exclusion criteria for participants participating in the daily at-home DBS collection study in Katakwi District, Uganda.

**Additional file 2: Table S2.** Comparison of mean TBP CT values for DBS collected at various times and locations.

**Additional file 3: Figure S1. **Correlation between the log_10_ *Pf* 18S rRNA venous blood and DBS samples collected during weekly clinic visits. **A** includes all sample pairs but uses volume-adjusted copy numbers for DBS; **B** restricts analysis only to pairs in which the DBS had 50 µL of blood.

## Data Availability

The datasets used and/or analysed during the current study are available from the corresponding author on reasonable request.

## Abbreviations

CI: Confidence interval
CT: Cycle threshold
DBS: Dried blood spot
LoD: Limit of detection
mRNA: Messenger ribonucleic acid
NARC: National HIV/AIDS Research Committee
*Pf*: *Plasmodium falciparum*
qRT-PCR: Quantitative reverse transcription polymerase chain reaction
RDT: Rapid diagnostic test
rRNA: Ribosomal ribonucleic acid
SD: Standard deviation
TBP: TATA binding protein
UNCST: Uganda Council for Science and Technology
UW: University of Washington
