# Supplementary material for: Feasibility of community at-home dried blood spot collection combined with pooled reverse transcription PCR as a viable and convenient method for malaria epidemiology studies
Source: Malar J. 2022 Jul 14;21:221. doi: 10.1186/s12936-022-04239-x (PMC9284728; doi:10.1186/s12936-022-04239-x)
Supplement: Supplementary file 1 — Additional file 1: Table S1. Full inclusion and exclusion criteria for participants participating in the daily at-home DBS collection study in Katakwi District, Uganda. [file 12936_2022_4239_MOESM1_ESM.docx]

**Table S1.** Full inclusion and exclusion criteria for participants participating in the daily at-home DBS collection study in Katakwi District, Uganda.

| **Inclusion Criteria** | - Males and female children aged 8-17 years old (with assent procedures) and male and female adults 18-60 years - Asymptomatic for Grade 2 or higher malaria-related signs and symptoms and afebrile (<38.0°C) at baseline - Lives in one of the seven villages in Katakwi District, Uganda - Does not plan to move out of the study area for one month following enrollment - Willing to self-collect a daily blood spot sample for 28-days (+/- 2 days) - Willing and able to return to the study clinic on a weekly basis to turn in DBS cards and provide 5 mL (adult) or 1 mL (child) of blood through venous draw at each visit - Reliable access to the clinical sites and availability to participate for duration of study - Able to fully understand the implications of study participation and provide informed consent - Able to provide assent (for child participants) - Agreement to come to the study clinic if study participant experiences febrile illness or prick site infection during the study period - Agreement not to take anti-malarial medications unless through the Malaria Clinic at St. Anne Health Center III - Agreement not to take other medications without informing the study team. - Absence of any significant chronic disease |
| --- | --- |
| **Exclusion Criteria** | - Being pregnant at enrollment or planning to get pregnant during the study period - Currently taking antimalarial treatment(s) - RDT-positive at screening - Any other finding that, in the judgment of the investigator, would interfere with, or serve as a contraindication to, protocol adherence, assessment of safety, or a subject’s ability to give informed consent, or increase the risk of having an adverse outcome from participating in the study |
