# Supplementary material for: Feasibility of community at-home dried blood spot collection combined with pooled reverse transcription PCR as a viable and convenient method for malaria epidemiology studies
Source: Malar J. 2022 Jul 14;21:221. doi: 10.1186/s12936-022-04239-x (PMC9284728; doi:10.1186/s12936-022-04239-x)
Supplement: Supplementary file 2 — Additional file 2: Table S2. Comparison of mean TBP CT values for DBS collected at various times and locations. [file 12936_2022_4239_MOESM2_ESM.docx]

**Table S2.** Comparison of mean TBP CT values for DBS collected at various times and locations

| **Group** | **n** | **Mean TBP CT (SD)** | **Difference (95% CI)** |
| --- | --- | --- | --- |
| Home spots | 1466 | 35.1 (1.2) | *(ref)* |
| Clinic spots | 314 | 34.3 (1.1) | -0.85 (-0.98, -0.71) |
|  |  |  |  |
| Week 1 | 374 | 35.2 (1.4) | *(ref)* |
| Week 2 | 375 | 35.1 (1.1) | -0.09 (-0.09, 0.27) |
| Week 3 | 365 | 35.2 (1.1) | -0.02 (-0.16, 0.2) |
| Week 4 | 365 | 35.1 (1.2) | -0.08 (-0.1, 0.27) |
|  |  |  |  |
| First day of week | 244 | 35.2 (1.1) | *(ref)* |
| Last day of week | 244 | 35.1 (1.2) | -0.01 (-0.2, 0.22) |
