# Supplementary material for: Feasibility of community at-home dried blood spot collection combined with pooled reverse transcription PCR as a viable and convenient method for malaria epidemiology studies
Source: Malar J. 2022 Jul 14;21:221. doi: 10.1186/s12936-022-04239-x (PMC9284728; doi:10.1186/s12936-022-04239-x)

**Figure S1.** Correlation between the log_10_ *Pf* 18S rRNA venous blood and DBS samples collected during weekly clinic visits. **(A**) includes all sample pairs but uses volume-adjusted copy numbers for DBS; (**B**) restricts analysis only to pairs in which the DBS had 50 µL of blood.


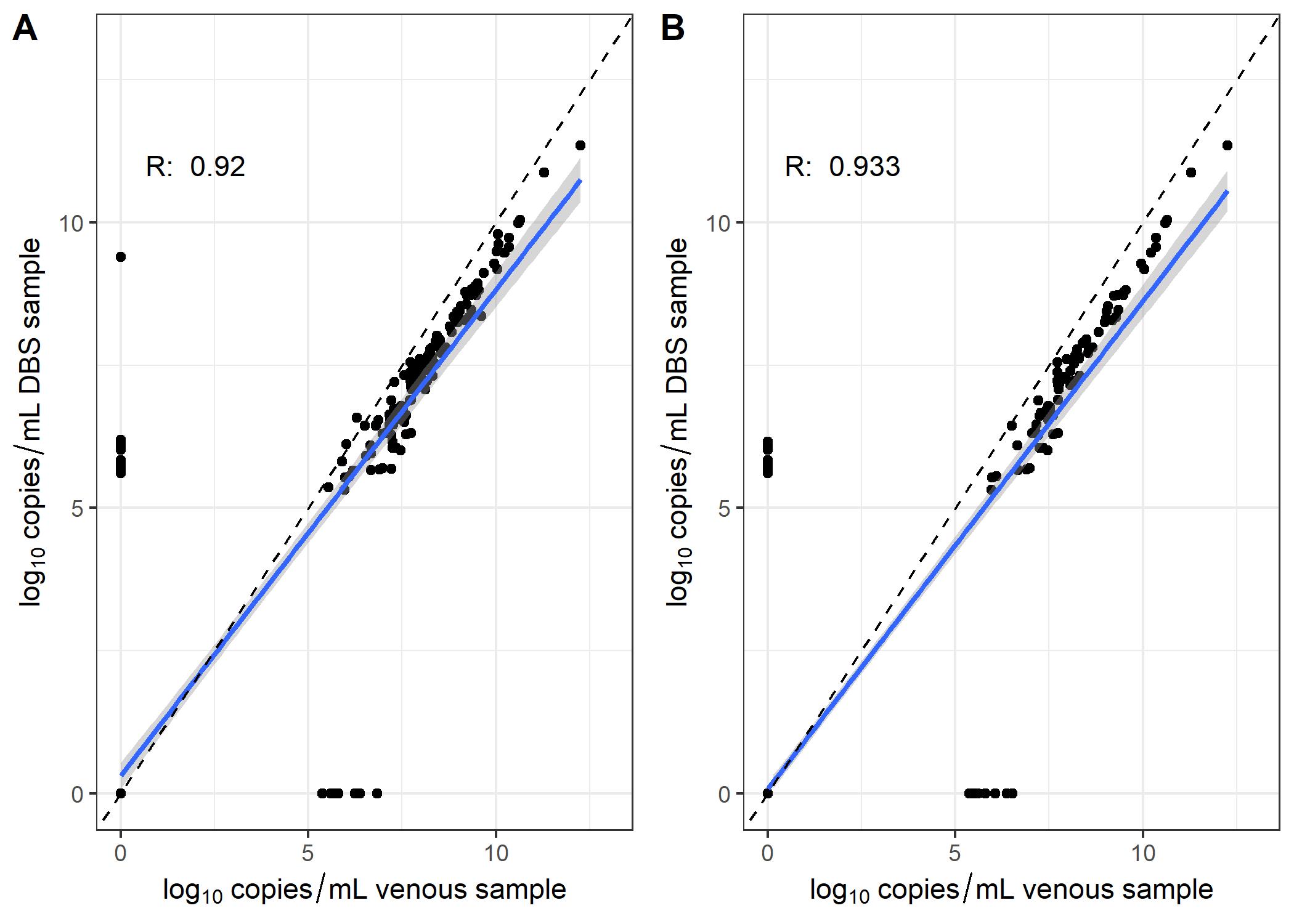

Supplement: Supplementary file 3 — Additional file 3: Figure S1. Correlation between the log10 Pf 18S rRNA venous blood and DBS samples collected during weekly clinic visits. A includes all sample pairs but uses volume-adjusted copy numbers for DBS; B restricts analysis only to pairs in which the DBS had 50 µL of blood. [file 12936_2022_4239_MOESM3_ESM.docx]
